# Adoption of change in endodontic practice after an educational program: A qualitative study

**DOI:** 10.1002/cre2.542

**Published:** 2022-02-18

**Authors:** Eva Wolf, Kerstin Leonard, My Vidigsson, Åke Tegelberg, Margaretha Koch

**Affiliations:** ^1^ Department of Endodontics, Faculty of Odontology Malmö University Malmö Sweden; ^2^ Department of Orofacial Pain and Jaw Function, Faculty of Odontology Malmö University Malmö Sweden

**Keywords:** implementation, rotary endodontics

## Abstract

**Objectives:**

The aim was to define the characteristics of successful implementation of new clinical endodontic routines within a public dental health organization, following an educational program.

**Materials and Methods:**

Fifteen staff members were strategically selected for the interview. All had completed a theoretical educational intervention including a complementary endodontic treatment strategy and, for the dentists, comprising training in the nickel‐titanium‐rotary‐technique. All experienced the successful acceptance of new clinical routines. Two thematic in‐depth audiotaped interviews were conducted, wherein the informants described the implementation process in their own words. The interviews were transcribed verbatim and analyzed according to Qualitative Content Analysis.

**Results:**

A theme was identified: *A multiple flexible process with governance support and gradual reinforcement of motivation*, with the following main categories: Firstly, *contextual facilitation, with two subcategories (i) a multifaceted organizational foundation* and *(ii) a tolerance of flexibility*. Secondly, *emotional facilitation*, with two subcategories *(i) an experience of simplification and (ii) an experience of improvement*.

**Conclusion:**

The results improve the understanding of a multifaceted process underlying the acceptance of changes to clinical endodontic procedures by dentists in a public dental health organization. Important contributing factors identified were governance support, a committed resource person with contextual knowledge, tolerance of flexibility in implementation, and permissive informal communication channels within the local workplace. These findings might be a valuable contribution to an evidence base, facilitating the selection of the most appropriate educational strategy and structure for a specified purpose.

## 1 INTRODUCTION

Endodontic treatment entails chemo‐mechanical cleaning of the root canal system: the aim is to remove microorganisms and necrotic or inflamed pulp tissue. Today, instruments made of nickel‐titanium alloy (manual, rotary, reciprocating, vibrating) are mainly recommended for this procedure (Cheung & Liu, 2009; Metzger et al., 2010; Molander et al., 2007; Pettiette et al., 1999,  2001). There are ongoing technological innovations, particularly of machine‐driven instruments. Different types of heat, polishing, and surface treatment of the alloys have led to the development of material appropriate for canal shaping. This is intended primarily to maintain the curvature of the root canal, optimizing cleaning of the canal walls (Pinheiro et al., 2018), but also to improve resistance to instrument separation (Basheer Ahamed et al., 2018).

Although there is, to our knowledge, no published evidence that one nickel‐titanium instrumentation technique is superior to another, in terms of the outcome measures healthy periapical tissues and tooth survival, the prognosis is good, provided cleaning, shaping, obturation, and coronal restoration are adequate (Dawson et al., 2016). However, machine‐driven canal preparation is reported to be quicker than manual instrumentation (Bjørndal & Reit, 2005; Peralta‐Mamani et al., 2019) and there progressive transition to machine‐driven instrumentation has been reported (Albuquerque et al., 2019; Koch et al., 2009; Reit et al., 2007).

It is sometimes desirable or necessary to introduce an evidence‐based change to clinical practice. Implementation strategies have been defined as “methods or techniques used to enhance the adoption, implementation, and sustainability of a clinical program or practice” (Powell et al., 2015) or as a “purposeful procedure to achieve clinical practice compliance with a guideline recommendation” (Mazza et al., 2013). Many strategies have been proposed to promote the implementation of knowledge in healthcare practice (Lokker et al., 2015). As in other disciplines, implementing change in clinical dental procedures can be a slow and unpredictable process (Bonetti et al., 2006; Clarkson et al., 2008), and a number of factors affect whether the change is accepted or not (Leeman et al., 2017). There are few publications in the dental literature exploring the reasons underlying successful adoption, failure to adopt, or rejection of new technology in clinical practice. In a public dental health organization, for example, successful implementation of new clinical routines and treatment approaches may require not only more knowledge of the educational design but also a greater understanding of the influence of contributing factors. This would allow the design of interventions with optimal effectiveness (Fixsen & Blasé, 2020; Mitton et al., 2007). In this context, the collection and analysis of qualitative data would be an important contribution (Burnard et al., 2008).

It is argued within hermeneutics that science cannot be reduced to causal explanations but that science is also an interpretation of the meaning in texts, actions, attitudes, and situations. It is essential to understand the meaning man puts to his/her actions, and by placing phenomena in an external context, they are better explained and understood. One seeks depth in the form of variety, nuances, and diversity rather than breadth in the information (Ricoeur, 1976).

The aim was to define the characteristics of successful implementation of new clinical endodontic routines within a public dental health organization, following an educational program.

## 2 MATERIALS AND METHODS

### 2.1 Qualitative content analysis (QCA)

The present study was conducted according to QCA, with an inductive approach and a focus on the informants and the context (Graneheim & Lundman, 2004). Content analysis is a scientific method used to extract and draw conclusions about the content of various types of communication and can be used for a variety of purposes, such as describing experiences, attitudes, or decision processes. It comprises both a quantitative and a qualitative approach and can be used inductively or deductively. The inductive approach involves analyzing data with little or no predetermined theory or framework. This approach has been suggested as appropriate when only limited knowledge is available about the study phenomenon (Burnard et al., 2008). When the empirical material comprises text from verbatim transcribed interviews, QCA analysis involves the researchers to repeatedly read the text, for him/her to get a sense of the whole. This is followed by a division of the text into meaning units, that is, divisions are made in the text at points a change in meaning occurs. The meaning units are condensed into more succinct formulations by excluding unnecessary words but with preservation of the essence. The condensed meaning units are then coded; that is, a label covering the core of significance is set. The codes sorted into subcategories and categories cover the manifest (descriptive) content. Finally, the emergence of a theme covering the latent (underlying message) content can be seen as a process that takes place throughout the analysis work and which stands as a summary result of the study. The categories and the identified theme together illustrate the pattern found (Graneheim & Lundman, 2004).

### 2.2 Context

The swedish public dental health service (PDHS) was founded in 1938. Clinics are located throughout the country and financed by a combination of taxes and patient fees (Koch, 2014). According to Swedish legislation (SFS, 1985), the aim of Swedish dental care is to ensure good oral health for the population and equality of access to dental care. Dental care must be of high quality and meet the patient's need for confidence in the provision of care and treatment. Approximately 40% of the Swedish population is treated by the PDHS (Tandvårds‐och läkemedelsförmånsverket, 2020).

In 2003 to 2004, an endodontist (MK) conducted an educational program supporting the implementation of rotary endodontics and a considered, rational approach to endodontics, in a regional PDHS organization. A total of 400 individuals participated in the program: dentists, dental hygienists, dental nurses, and administrative personnel. The purpose of the training program was to implement a rotary nickel‐titanium‐based instrumentation system, ProFile® (Dentsply Maillefer, Ballaigues, Switzerland), single cone obturation of the root canals, and to streamline routines for endodontic schedule planning, emergency treatment principles, and asepsis (Koch et al., 2009).

The educational program, which was supported by the governance of both the regional PDHS and all the attached clinics, included:

2.2.1

• A personal visit by the endodontist to introduce the program at all clinics
  ➤ the dentists: full‐day training comprising both theory and practical exercises on plastic models and extracted teeth
  ➤ other staff: 2 h of theoretical education as a knowledge base
• The endodontist and the assistant staff were available for discussion during the training day
• Subsequent feedback and recurring practical recommendations were available by e‐mail contact with the endodontist.

### 2.3 Subjects

Fifteen informants (Table 1) were, among seven clinics, strategically selected according to the following criteria:

- Had participated in the endodontic educational program described above;
- Were still working in clinics where the new clinical routines and NiTiR techniques had been implemented.

**Table 1:** Description of the informants: professional role, duration of professional experience, affiliation with clinic which participated in the educational course, sex, and age

Informant	Role (*N*)	Duration of professional experience (year)	Clinic affiliation (*N*)	Sex	Age (year)
1	Dental nurse^a^ (3)	18−27	2, 5, 7	Female (3); male (0)	46−65
2	Dentist (8)	6−36	3, 4, 5^3^, 6^2^, 7	Female (6); male (2)	30−64
3	Dentist, clinical manager (4)	20−30	1, 2, 5, 7	Female (1); male (3)	48−60

^a^ Dental nurse; dental nurse, receptionist; dental chief nurse.

Following written information about the study, they were invited by the interviewer (MK), also the educator, to participate. The potential informants had diverse occupations: clinical managers, general dental practitioners (GDPs), dental assistants, or receptionists. There was diversity of sex, occupational experience, and present location. The informants represented 7 of the 16 clinics that had participated in the educational program. All those invited consented to participate.

### 2.4 Data collection

During 2010, 30 thematic in‐depth interviews (two with each informant by MK) were conducted after an initial mail contact with the clinical manager followed by a mail to the informant in question. The location for the interview was the informant's clinic with one exception, where a restaurant was chosen. The interview technique encouraged the informants to describe in detail, in their own words, the introduction of the new endodontic routines and techniques at their clinic. The digitally recorded interviews were conducted mainly with open‐ended questions, but more direct follow‐up questions were asked, to encourage further development and concretization of the narrative. The first interviews lasted for 25−60 min and the second for 12−39 min. After the first interview, the informants received a copy of the audio file. The interviews were transcribed verbatim, including notations of nonverbal expressions.

### 2.5 Data analysis

**Figure 1:**
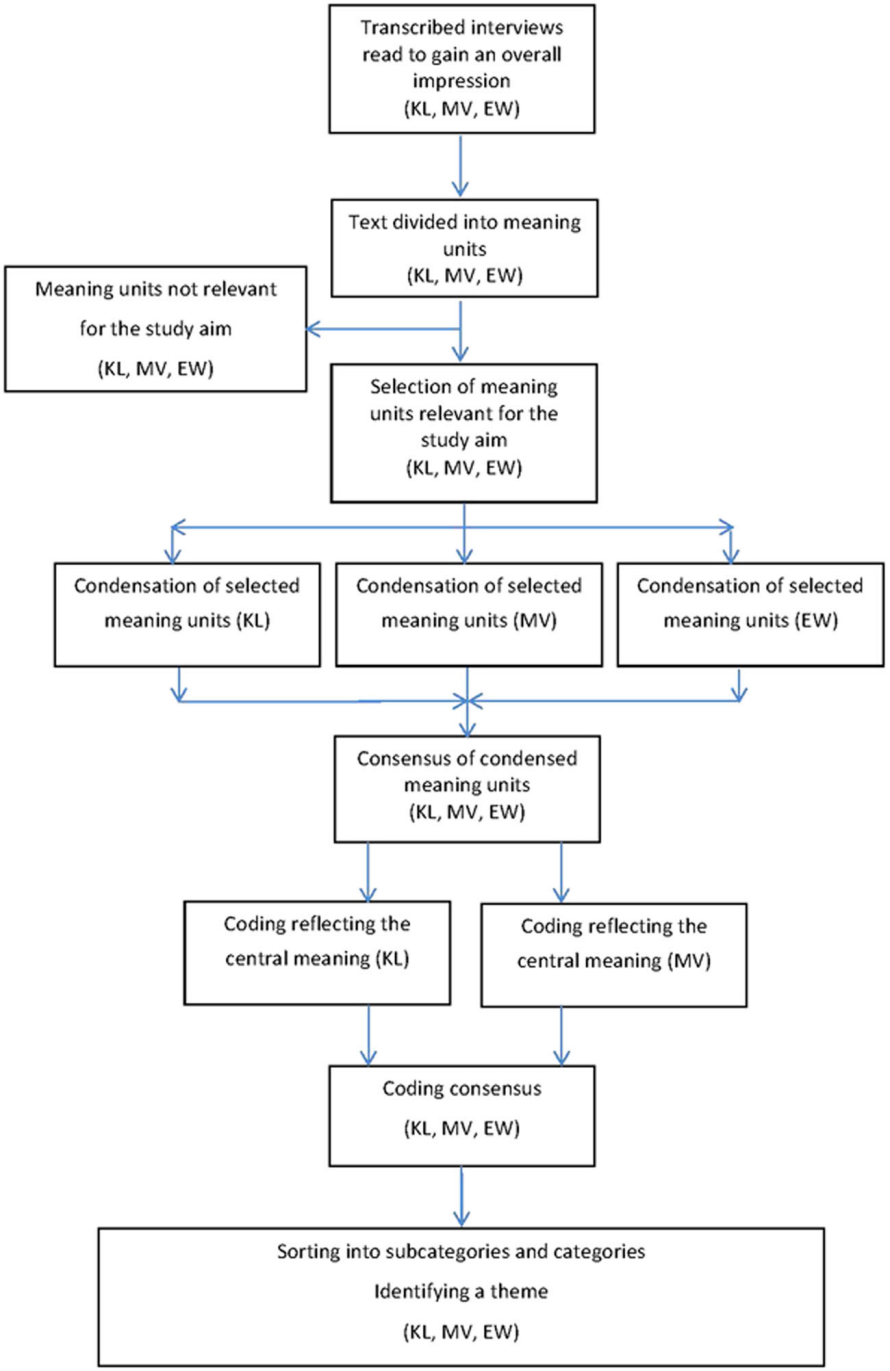
Flowchart illustrating the analysis process of the collected interview material

**Figure 2:**
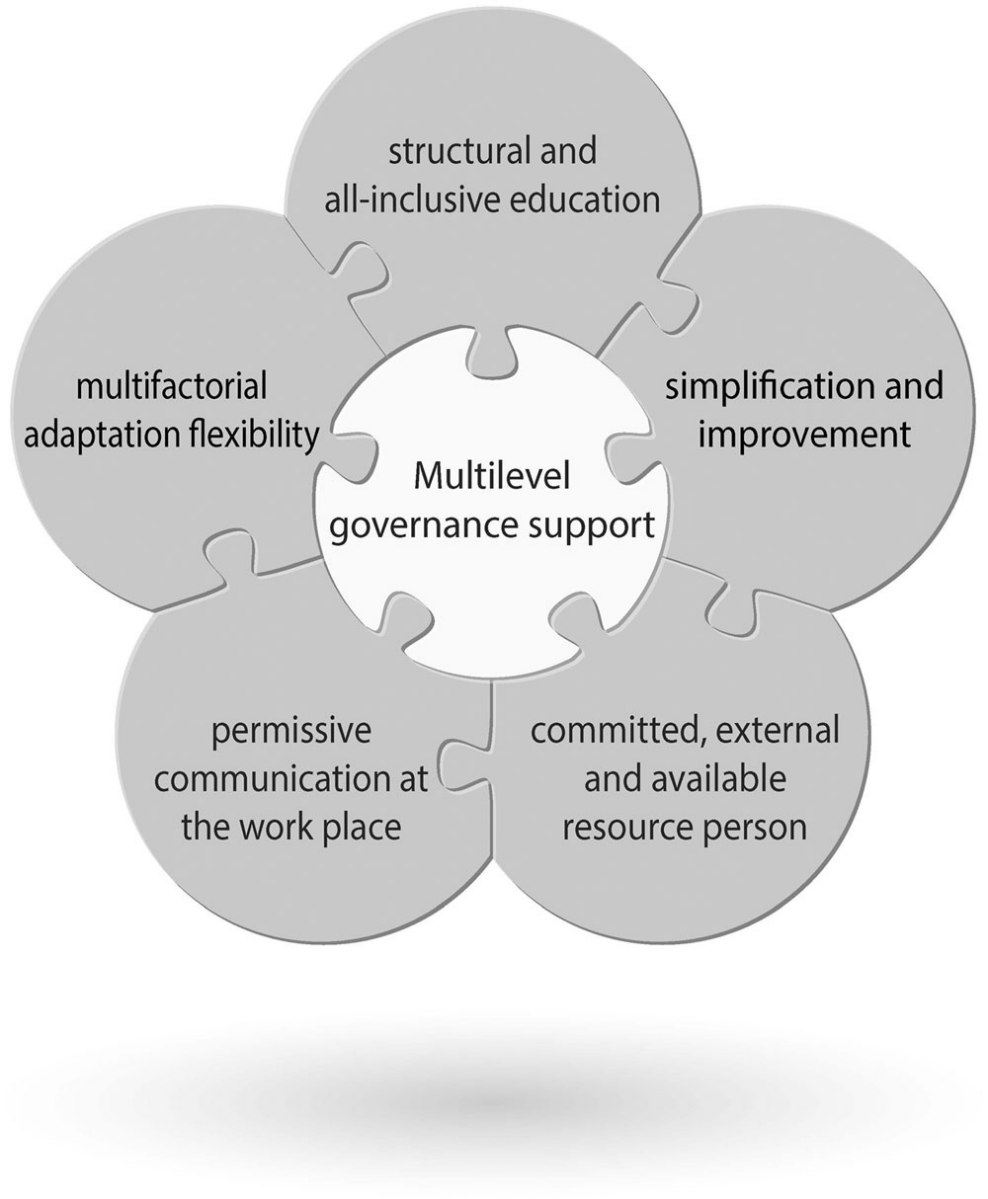
Illustrating the theme identified; a multiple flexible process with governance support and gradual reinforcement of motivation

The qualitative content was analyzed according to Graneheim and Lundman (2004) (Table 2, Figure 1). The codes were sorted into subcategories and categories representing the manifest content. An overall theme was identified, that is, an interpretation of the underlying message, representing the latent content (Figure 2). The informants did not comment on the transcriptions or provide feedback on the findings. The consolidated criteria for reporting qualitative research (COREQ) checklist was completed.

**Table 2:** Qualitative content analysis process used to analyze interviews and extract results. Two meaning units condensed into more succinct formulations, that is, condensed meaning units are visualized with the corresponding code, subcategory, and category

Meaning unit	Condensed meaning unit	Code	Subcategory	Category
Having you here, it meant such a lot that you… Had expectations of us, so that we would give it a try. We should have teeth to work on eh, that we should… that you came back later and followed up, it meant a great deal. They, yes in some way it was taken more seriously then. But you didn't let up either. It was in fact something like this: “Here I come,” “Now you are to do this,” “By next time you are to have tried.” And … yes.	It meant a lot having you here, had expectations of us. “By the next time you are to have tried.” It was taken seriously then.	Committed resource person	Multifaceted organizational foundation	Contextual facilitation
I remember that it felt as if, it…., it would SIMPLIFY the working day if one had just ONE system. Because we had so many different sorts of files…, everything from small files to Hedströms, yes, S‐files and. (sighs). Oh yes, handling in the steri room and eh, purchasing also. That one only had this now. Before, so, there were so many different items one had to keep track of.	It would simplify if one had only ONE system. Before there were many different items to keep track of	One system simplifies	An experience of simplification	Emotional facilitation

### 2.6 Ethical considerations

The study was conducted in accordance with the 1964 Declaration of Helsinki II (version 2002 revision, www.wma.net) and was approved by the Ethics Review Board at Karolinska Institute, Dnr 2008/1723. The informants received verbal and written information about the project and gave written informed consent. When a new principal investigator and new researchers joined the study, a supplementary application was made in 2017 to the Ethics Review Board at Lund University and was approved, Dnr 2017/476.

## 3 RESULTS

The overall theme illustrating the latent content was *a multiple flexible process with governance support and gradual reinforcement of motivation* (Table 3, Figure 2). The pattern identified comprised top‐down management support combined with an individualized learning process, supported by encouragement and characterized by the participation of the course participants.

**Table 3:** The identified pattern found, illustrated by the theme, categories, and subcategories

Theme	A multiple flexible process with governance support and gradual reinforcement of motivation	
Category	*Contextual facilitation*	*Emotional facilitation*
Subcategory	A multifaceted organizational foundation	An experience of simplification
	A tolerance of flexibility	An experience of improvement

Two categories illustrating the manifest content were identified. Firstly, the category *contextual facilitation* with the two subcategories (*i*) *a multifaceted organizational foundation* and (*ii*) *a tolerance of flexibility*. Secondly, the category *emotional facilitation* with the subcategories (*i*) *an experience of simplification*, and (*ii*) *an experience of improvement* (Table 3).

### 3.1 Contextual facilitation

This category identified the following advantageous contextual factors: governance support throughout the organization, the design of the educational program, and acceptance of flexibility in implementation.

The first subcategory, *a multifaceted organizational foundation*, described the importance of how the decision to introduce a change to clinical practice was accomplished, as well as how the educational program was designed with both depth and width.

The importance of regional organizational support, firmly established in the clinical core function, involving the entire organization, and grounded in an overall strategy as well as a positive attitude to change, was expressed as beneficial to the success of the implementation.

I think it matters a lot that ehh… …, matters more than you'd think, I'd say, that if it is something that is said in some sort of strategy decision, that now we're going to DO this, the whole organization. And everyone knows this, from the most senior executives down. This is how it is, it's been decided and now this is how we are going to do things (Informant 6).

The importance of comprehensive governance support was also mentioned, in contrast to when a single individual tries to initiate a change.

Perhaps one of us heads off…, one or two of us to a course and then well, ehh, then you come home and are enthusiastic but, but to get all the others interested, that's actually really difficult (Informant 2).

The informants spoke of the importance of the design of the educational program, with a clear structure and involving the entire organization of the clinic in question.

There was a structure ehhhh, and most importantly a well‐supported background as to WHY one should work in this way, and that in fact included the entire concept. It is not just about machine‐driven reaming, but how we manage root canal treatments. Everything from the emergency patient to, to ehh, the actual technique for how you clean the canal and to have that understanding as backup. Obviously it is much simpler and this motivates both one‐self and ehh, the rest of the team as to why one should work this way (Informant 6).

The educational program was designed with both theoretical and practical components, comprising an easily understood presentation of the scientific background, and practical information. The informants appreciated oral instruction, but also the opportunity to test the new technique practically (on extracted teeth), under supervision, before chairside application.

There are so few things I have changed so ABSOLUTELY in our, my way of working hm, as with endodontics, so I was surprised that I was so… positive, so quickly. […] What really surprised me was that I felt in fact that I had actually got so much information beforehand, that I dared to give it a try (Informant 4).

The informants described the commitment and encouragement of the resource person as beneficial, and also appreciated the fact that she made demands of the participants. Having the resource person present and available throughout the process, with both theoretical input and practical exercises, was considered encouraging. The subsequent feedback was expressed as valuable and a contribution to the successful implementation.

Oh yes, it is in fact good that if one had any problems or the like then one could in fact ring you (Informant 4).

The informants appreciated the fact that the educator was external to the clinic, but working within the same regional organization and thus had contextual knowledge. The trustworthiness of the information provided was reinforced by the fact that the educator was a consultant in endodontics and thus a source of both theoretical and practical knowledge.

It felt very good to have someone from outside (the clinic). It added a little weight to it too, particularly when it was a specialist (laughs)… It has meant a lot (Informant 1).

We have the same culture, this study culture which eh yes in fact we understand each other, each other's workaday life… and hm, that means I can rely a little more on what you say, that's obvious (Informant 15).

The implementation process also included organizational support for the new routines, to be discussed at clinic meetings.

We like to talk things over among ourselves first so we have fewer issues to tackle. And it's the same with the steri room and instrument trays and so on, so we would really like……. to have standardized instrument trays, so everyone gets the same, to simplify processing. And, and we are also very strict about not having too many things on the trays either so that…. We talk things over and remove instruments and so on (Informant 15).

The informants emphasized the importance of permissive communication channels within the clinic, characterized by a social climate of cooperation, with the opportunity of having an impact during the process and also collegial support.

Then naturally, the combination of people, too, that they are used to, …., adapting and…., trying new things and adapting after discussion with one another(Informant 11).

There were one or two dentists here… who had already started and ehh, had taken on just such a single‐rooted tooth and thought it was this way or that and gave us a few tips (Informant 7).

A sense of general inclusiveness was expressed as beneficial. All occupational categories had access to the same guidelines. This was also perceived as transparent: everyone was invited to participate and become familiar with the new clinical routines.

The second subcategory was labeled *a tolerance of flexibility*. The educational program was compulsory, but each individual was able to decide, voluntarily, when and also to what extent they would adopt the new routines.

I think it's important that one isn't pushed into doing something, but can decide for oneself when to start, and so on. Because there are in fact always some people who are quick and competent, but then there are also those who are competent but not as quick, so that it… (Informant 8).

We have introduced this to, to new staff when they are appointed. A number of dentists have joined the staff since we introduced this to the clinic and and eh we have in fact introduced this (the implementation) quite deliberately to those who have come to work here since, and explained that this is the clinic's policy on root fillings, and eh we haven't forbidden them (laughs) to clean manually, but as far as I know I believe that everybody uses it today (Informant 2).

It was recommended that the guidelines be followed routinely, but in isolated cases it was still permitted not to do so if it was not possible to apply the new method. There was also variation and flexibility in regard to the attitude to change. Informants brought to light personal preferences, like being keen on or skeptical about new interventions, or not discouraged by initial problems when adapting to a new routine. A further motivation for changing to the new method was lack of complete satisfaction with the results and quality of the former, familiar method.

You asked why one would want to change eh, and… the answer is in fact that one is not completely satisfied with what one has been doing. Because if one thinks it is perfect… well then one wouldn't want to change (Informant 12).

Informants also commented that there had been a few too many hopes dashed over grand revolutions in dentistry and that this may explain why one is at first skeptical about accepting change, preferring to wait until others have sorted out the mistakes and thereafter to decide for oneself.

### 3.2 Emotional facilitation

This main category covers a pattern elucidating the importance of experiencing benefits from several perspectives, such as simplification and improvements, not only during the educational program but also during subsequent clinical application.

*An experience of simplification* was obvious and frequently expressed during the interviews, meaning both adoption and maintenance of working methods and guidelines. The overall instrumentation, comprising the entire process including the number of instruments being used, was described by the informants as simplifying factors.

When the nurse came and said like: “Oh, AT LAST” (laughs). Because they also thought that, they're (the ones who) put out the instruments and keep an eye on things, hm. This was so SIMPLE because here you knew exactly (what was needed) (Informant 14).

The informants also stated that the new way of working not only facilitated the work of the dentist but had a positive effect on the function of the whole clinic. That the new routines were simplified the work of personnel in the steri room and the dental nurse also meant that the dentist's work flowed more easily.

Then there was really good support from the nurses, because they thought that the instrument trays were disorganized and there were issues with sterility. One had to fetch files and the file rack didn't function well and so on. And processing instruments in the steri room was difficult after manual cleaning of the root canal because the file rack hadn't been used but a whole lot of extra files had been used instead, but the file rack still had to be dealt with then. So there was huge support and I believe that was important (Informant 12).

Motivation was gradually reinforced as it became apparent that the new technique was actually quicker and easier.

That one discovered so soon that it was in fact quicker and simpler. Then one is in fact motivated. Yes. It is like ehhh, in fact ehh, root fillings are really the sort of thing that some, or many dentists think: “Oh no, what a bother”, eh (Laughs). But, but in fact when you start with this you actually felt a little, yes, one felt a new inspiration to do root fillings (Informant 2).

The informants commented that even physical exertion decreased with the adoption of the new technique.

I think there is less strain on ehh, how you work. You sit much better ergonomically…. I think. Before I thought you strained fingers and hands such a lot when you were filing by hand… and then you kind of had pain in your hands afterwards and then perhaps, in the beginning when you were a new dentist, maybe you tensed up even more… just because you thought it was harder. But when this technique works so well, then you are more relaxed too and sit much better (Informant 5).

By comparison, the previous method was considered to be arduous and complicated. Experiencing the simplification reinforced motivation to adopt the new routine. The new technique was also easier and systematic and the informants observed that it did not cause any increased frequency of unwanted complications such as file separation.

It is simpler. It isn't in fact, there are actually not so many instruments, in fact there aren't… Before you would sit there and hack away and test how far you had got with some files. Now one is like more systematic according, according to this system, eh and it, it works well in most cases…, I think. What hasn't been so good is that I have had some file fractures, but that happened in fact with, with manual cleaning too. Yes, … So they, they are the ones which haven't been successful (Informant 2).

Not all routines were changed for the informants but they stated frequently that the new routine facilitated work planning for the appointment. Another benefit expressed was that in practice the method matched the information provided.

Because a method like this that actually works in the way it is described, that makes it easier to accept it (Informant 9).

The informants expressed that the new routines and techniques offered some kind of security, given that for all involved, the new structure was easy to follow.

The second subcategory, *an experience of improvement*, included a number of different factors that apply to both time spent/economy, quality, and personal satisfaction.

Different aspects of the economy were mentioned, including fewer patient visits, less time spent, fewer instruments used, all resulting in increased income for the clinic.

Just the fact that there is only…., you need so few appointments (Informant 3).

I can imagine that our financial manager was also in support of this…. And this idea of including EVERYONE, eh. Treatment times were shorter, so we could treat other patients in that time. The whole of it was in fact like… Yes, that you SAVE money when you look at the final outcome (Informant 14).

The former method was also considered to be more unpredictable in comparison. There was agreement within the clinic that the guidelines should be followed in order to create satisfactory circumstances. With the new method the root filling actually often, although not always, ended up where it was supposed to end up and subsequently with a better result.

But the root fillings are better. I mean better quality (Informant 13).

It was a bit unpredictable before…. and it might even be like that now, I won't say that. It sounds as if you are always successful, and that's not the case… but it feels safer […] It is such a satisfying feeling when one achieves a good result. That is just how it is (Informant 8).

Also mentioned was a sense of satisfaction in mastering a treatment procedure that some colleagues found difficult.

It does in fact give you a feeling of satisfaction to be able eh, to be able to master a treatment procedure which nevertheless is considered to be relatively hard to do. Eh, to feel that one has mastered it well, that gives you a personal sense of satisfaction. It does in fact…, undoubtedly (Informant 2).

Personal satisfaction among the dentists was important and linked to an expressed sense of joy in having the ability to follow a structured procedure, reinforcing professional confidence, both when it comes to knowing what to do and doing it with a good result.

As a professional, well, one has. There is a kind of pride and, and the fact that I…. I think, in any case, I must say that I think I am quite competent at these things. “And now I have achieved this, so if I now do something new then can I consider myself competent”? Eh, I need to see for myself, that in fact I can handle these new things and the result is good…. and that it…., then if we are to change our treatment methods too. “Be better?” That is in fact what one wants (Informant 6).

The new technique was considered a source of satisfaction not only for the working staff but also for the patients. The informants considered it positive that the treatment they were providing also gave their patients a better standard of care.

It's relaxed, there is less stress, it is the patients…., it's better for the patients too in that it is less stressful. Ehh, because everything one has planned goes ahead as, as it is meant to. Hopefully this makes it less stressful for the patients too (Informant 10).

The informants also commented on the environmental benefits of a simplified system, with fewer instruments and equipment and minimum wastage.

## 4 DISCUSSION

The analysis facilitated an improved understanding of factors determining successful clinical change. Important contributing factors included governance support, a committed resource person with contextual knowledge, followed in the implementation stage by tolerance for flexibility in adopting the new technique and encouragement of open communication at the local clinic level.

A scientific method for analysis should be chosen with regard to the framing of the scientific issue to be investigated. To address people's subjective understanding of a phenomenon and explore factors influencing acceptance of a clinical change, as in this case, the collection of qualitative data was considered appropriate (Pope & Mays, 2020) and a corresponding scientific method of analysis was chosen (Graneheim & Lundman, 2004). The strategy of using semi‐structured interviews and mainly open‐ended questions, together with analysis after collecting the entire material, meant that the interviewer could avoid own preconceptions during the interview and could also follow and not direct the informant. Thus the interviewer's impact on the narration was minimized (Miles & Huberman, 1994). To strengthen credibility (Miles & Huberman, 1994), the coding was done independently by two authors (K. L., M. V.) and then discussed (K. L., M. V., E. W.) (Figure 1). To improve the credibility criteria further, representative quotes are presented as examples of the pattern found.

The informants were interviewed twice and after the first interview had the opportunity to reflect on opinions and statements. This allowed the informant to correct and clarify their narratives. As the interviews were conducted by the educational resource person, there is some potential risk of bias. During the interview, the informants might have been reluctant to describe possible negative experiences. However, this shortcoming might be of limited importance because the interview was focused on factors that facilitated implementation and not on detrimental factors.

The richness of information in the interviews is critical, as it will determine how saturation is achieved. Saturation also depends partly on the number of informants (Boddy, 2016). In this study, 15 informants were strategically selected, all with documented experience of the phenomenon to be studied (see criteria above). Moreover, all were interviewed twice. This reduced the risk of failure to achieve saturation. As the informants represented different occupational categories with a diversity of experiences, they presented a range of perspectives. Although full saturation might still not have been achieved, this does not necessarily invalidate the findings but indicates that the topic has not yet been fully explored (Boddy, 2016; Morse & Field, 1995). Rather than presenting definitive answers, the results contribute to providing indications and a broader understanding of factors of importance for the implementation of new clinical routines and practice and improved the potential for transferability, at least to similar contexts.

With respect to validity, the findings were compared with those of a study (Koch et al., 2014) using the descriptive phenomenological human scientific method (Appelbaum, 2012; Giorgi, 2009). The same phenomenon of successful change was investigated in eight interviews with four of the 15 informants in the present study. Although expressed somewhat differently (due to the different method of analysis), essentially similar experiences of the meaning of change in clinical practice were revealed. In the phenomenological study, (1) motivating expectations, (2) the allowance for an individual learning process, (3) continuous collaboration, and (4) a facilitating educator, emerged as key constituents, all interdependently necessary to describe the structure of the phenomenon. These aspects correspond to the general findings in the present study.

The influence of the backing of all different management levels of the organization was relevant to successful change. The organizational priority was evident, and this has previously been shown to be important (Birken et al., 2015). Although the decision to introduce an educational program was made at the highest management level, including the middle managers (Urquhart et al., 2018) and involving all personnel (Koch et al., 2009; Lukas et al., 2007) in the suggested change, proved essential. Moreover, the option of individual flexible implementation rates and the positive experiences of various forms of improvement motivated the staff to continue with the new clinical routines and treatment approach. This facilitated discussion and a sense of participation. Apart from the importance of improving everyday work, the collaboration circumvented the sense of disempowerment described by the informants, of not being in a position to initiate change by themselves.

Scientific evidence per se as a motive for change was, as previously shown (Koch et al., 2009), considered important but analysis revealed that it was only one of several influential factors. Possibly of greater importance was that the informants considered the resource person to be a competent representative of scientific evidence, who was also capable of adapting to local conditions: that is, able to transfer structured knowledge both theoretically and practically. It has been shown that implementation is facilitated by an approach tailored and adapted to the current local target group, at individual and environmental levels (Forsner et al., 2010; Grol & Grimshaw, 2003). This is supported by the results of the present study, noted by informants from several perspectives and not least by identification and tolerance of multilevel flexibility.

It is reported that implementation relies on the suggested change being sustainable at a reasonable cost and not only credible but also feasible (Proctor et al., 2011). This is confirmed by the pattern found in the present study. The financial advantage was based on faster throughput of patients and savings on costs of materials (Hoomans & Severens, 2014), although the latter has been contradicted (Koch et al., 2012). There are currently no studies that clearly show one instrumentation technique to be superior to another, with respect to healthy periapical status or tooth survival as outcome measures (Methods of diagnosis and treatment in endodontics, 2012) Successful implementation in the present study might still be relevant from a health economic perspective. The routine and technique are reported to be faster (Peralta‐Mamani et al., 2019), although several studies have shown that rotary instrumentation does not have a better prognosis or tooth survival than manual instrumentation with nickel‐titanium instruments (Koch et al., 2015; Schäfer & Bürklein, 2012).

The impact of emotional factors should be considered as possibly important for implementation. Providers and staff were highly motivated, seeing the suggested approach as an opportunity to promote practice management. This is also in combination with improving clinical efficiency, productivity, and the generally expressed and permeating sense of simplification/improvement. The dentists/staff strive to provide high‐quality care but perceived that existing routines failed to achieve this. Here, a comparison was made with the previous quality of endodontic treatment, also previously reported to be of importance (Rycroft‐Malone et al., 2002). However, emotions certainly played an important role also regarding public health. This was due to the perception that the patients would benefit from the proposed new approach.

An evidence‐based system for innovation support (EBSIS) has been suggested by Wandersman et al. (2012). This system includes technical assistance and practical training, as well as quality assurance/quality improvement, all of which are shown in the pattern identified in the present study to be essential factors for encouraging implementation. Practitioners' capacity is also considered in studies of implementation of evidence‐based interventions (Collins et al., 2006; Rabin et al., 2010) where it is suggested that “capacity” be defined as “the provision of ongoing support for the purpose of increasing practitioners' awareness, knowledge, skills, self‐efficacy, and motivation to adopt and implement a new intervention” (Flaspohler et al., 2008). Capacity‐building interventions have previously been shown to be effective in the adoption/implantation of evidence‐based interventions (Durlak & DuPre, 2008; Flaspohler et al., 2008; Mitton et al., 2007), although to date the best design for maximizing effectiveness is still under discussion (Leeman et al., 2017; Mitton et al., 2007). Before designing an educational program, it seems to be important to reflect upon the reason and in what context the knowledge is intended to be implemented.

## 5 CONCLUSIONS

It is largely unknown which educational strategies are most effective in facilitating implementation by clinicians of new, evidence‐based clinical procedures. The results of this study illustrate the complex interaction of psychosocial and behavioral factors, as well as perceived benefits and advantages, which influence the successful chairside implementation of new technology. The findings might be an important contribution to the future synthesis of findings across studies, in order to build an evidence base, facilitating selection of the most appropriate educational strategy and structure for a specified purpose.

## CONFLICT OF INTERESTS

The authors declare that there are no conflict of interests.

## AUTHOR CONTRIBUTIONS

*Conceived the study and design, contributed to analysis, writing, editing, and critical review*: Eva Wolf. *Contributed to analysis and writing*: Kerstin Leonard and My Vidigsson. *Conceived the study and design, contributed to writing, editing, and critical review*: Åke Tegelberg. *Conceived the study and design, collected the material, contributed to writing, editing, and critical review*: Margaretha Koch. All authors gave final approval and agree to be accountable for all aspects of the work.

## Data Availability

Data available on request due to privacy/ethical restrictions.
